# Validation of SV2A-Targeted PET Imaging for Noninvasive Assessment of Neuroendocrine Differentiation in Prostate Cancer

**DOI:** 10.3390/ijms222313085

**Published:** 2021-12-03

**Authors:** Bing Guan, Ning Zhou, Cheng-Yang Wu, Songye Li, Yu-An Chen, Sashi Debnath, Mia Hofstad, Shihong Ma, Ganesh V. Raj, Dalin He, Jer-Tsong Hsieh, Yiyun Huang, Guiyang Hao, Xiankai Sun

**Affiliations:** 1 Department of Radiology, University of Texas Southwestern Medical Center, Dallas, TX 75390, USA; guanbing27@163.com (B.G.); ning.zhou@utsouthwestern.edu (N.Z.); cheng-yang.wu@utsouthwestern.edu (C.-Y.W.); sashi.debnath@utsouthwestern.edu (S.D.); 2 Department of Urology, The First Affiliated Hospital of Xi’an Jiaotong University, Xi’an 710061, China; hedl@mail.xjtu.edu.cn; 3 PET Center, Department of Radiology and Biomedical Imaging, School of Medicine, Yale University, New Haven, CT 06510, USA; songye.li@yale.edu (S.L.); henry.huang@yale.edu (Y.H.); 4 Department of Urology, University of Texas Southwestern Medical Center, Dallas, TX 75390, USA; yu-an.chen@utsouthwestern.edu (Y.-A.C.); mia.hofstad@utsouthwestern.edu (M.H.); shihong.ma@utsouthwestern.edu (S.M.); ganesh.raj@utsouthwestern.edu (G.V.R.); JT.Hsieh@utsouthwestern.edu (J.-T.H.); 5 Advanced Imaging Research Center, University of Texas Southwestern Medical Center, Dallas, TX 75390, USA

**Keywords:** neuroendocrine prostate cancer (NEPC), neuroendocrine differentiation (NED), synaptic vesicle glycoprotein 2 isoform A (SV2A), positron emission tomography (PET), ^18^F-SynVesT-1

## Abstract

Neuroendocrine prostate cancer (NEPC) is an aggressive and lethal variant of prostate cancer (PCa), and it remains a diagnostic challenge. Herein we report our findings of using synaptic vesicle glycoprotein 2 isoform A (SV2A) as a promising marker for positron emission tomography (PET) imaging of neuroendocrine differentiation (NED). The bioinformatic analyses revealed an amplified SV2A gene expression in clinical samples of NEPC versus castration-resistant PCa with adenocarcinoma characteristics (CRPC-Adeno). Importantly, significantly upregulated SV2A protein levels were found in both NEPC cell lines and tumor tissues. PET imaging studies were carried out in NEPC xenograft models with ^18^F-SynVesT-1. Although ^18^F-SynVesT-1 is not a cancer imaging agent, it showed a significant uptake level in the SV2A^+^ tumor (NCI-H660: 0.70 ± 0.14 %ID/g at 50–60 min p.i.). The SV2A blockade resulted in a significant reduction of tumor uptake (0.25 ± 0.03 %ID/g, *p* = 0.025), indicating the desired SV2A imaging specificity. Moreover, the comparative PET imaging study showed that the DU145 tumors could be clearly visualized by ^18^F-SynVesT-1 but not ^68^Ga-PSMA-11 nor ^68^Ga-DOTATATE, further validating the role of SV2A-targeted imaging for noninvasive assessment of NED in PCa. In conclusion, we demonstrated that SV2A, highly expressed in NEPC, can serve as a promising target for noninvasive imaging evaluation of NED.

## 1. Introduction

Prostate cancer (PCa) remains the second leading cause of deaths across all cancer types in American men [1]. Although the 5-year cancer-specific survival rate for patients with clinically localized and regional PCa is nearly 100%, the 5-year cancer-specific survival sharply drops to 31% for metastatic castration-resistant prostate cancer (mCRPC) [2]. Classified as neuroendocrine PC (NEPC) [3,4,5], a subset of mCRPC with universal neuroendocrine differentiation (NED) features manifests an even worse prognosis with an overall survival of <1 year [6,7]. The current therapeutic regimen for NEPC is limited to cisplatin or carboplatin in combination with taxanes, but shows minimal effects [3,8]. The predominant type of PCa, adenocarcinoma PCa (AdPC), has been documented with focal NED features (10% to 100%) [9]. A positive correlation has thus been suggested between NED and the poor prognosis in PCa [5,9,10], indicating that NED can potentially serve as a prognostic indicator for PCa.

Indeed, NED has been implicated in neuron–cancer interactions or innervations during the development and progression of PCa [11,12,13,14,15]. Both epithelial and PCa cells can undergo perineural invasion and adopt a true neural-mimicking phenotype [16,17], by which PCa cells circumvent the stressful situations resulting from androgen deprivation therapy (ADT) [5,18,19]. NED or NE-like cells produce and secrete a cocktail of mediators commonly seen in the nervous system, and these neuropeptides have mitogenic effects that sustain the growth and survival of adjacent cancer cells [5,20]. As such, NED may serve as a potential target to develop theranostic strategies for cancer with neuroendocrine (NE) phenotypes.

Current diagnosis of NED relies on the pathologic assessment of biopsies [21]. However, its accuracy is limited by sampling bias and the fact that repeated biopsy procedures are not clinically feasible. In addition, there is always a lag between the underlying molecular initiation and the phenotypic appearance. Given its noninvasive and quantitative features coupled with inherent superior sensitivity, positron emission tomography (PET) has become a molecular imaging tool revolutionizing the management of PCa, as evidenced by the approvals of Choline C-11 (12 September 2012) and Fluciclovine F-18 (Axumin^®^) (27 May 2016) from the United States Food and Drug Administration (FDA) for PET-imaging diagnosis of recurrent PCa, as well as the FDA’s most recent approval of ^68^Ga-PSMA-11 (1 December 2020) [22] and ^18^F-DCFPyL (Pylarify, 27 May 2021) for prostate-specific membrane antigen (PSMA)-positive lesions in men with PCa. However, none of the agents are of potential use to detect NED in PCa, as NE progression leads to downregulated choline metabolism [23], decreased proliferation rate [24], and reduced PSMA expression [25]. Due to its increased glycolytic activity, there have been revived interests in the use of ^18^F-FDG for NEPC [25,26,27]. Molecular mechanisms aside, the urinary excretion of ^18^F-FDG along with the generally low glycolysis in PCa presents a technical obstacle for ^18^F-FDG-PET to detect NED in PCa. The FDA-approved ^68^Ga-DOTATATE (NETSPOT™) for somatostatin receptor 2 (SSTR2) positive neuroendocrine tumors (NETs) has been long suggested to diagnose PCa patients with NED. However, recent case reports gave mixed results with wide tumor uptake ranges [28,29,30,31], in addition to observed intra-patient tumor uptake variations [30,31,32]. Obviously, noninvasive assessment of NED in PCa and other cancer types is an unmet clinical need.

In search for a target to develop NED-targeted theranostics, we have found that synaptic vesicle glycoprotein 2 isoform A (SV2A) can serve as a promising candidate. Currently, NED is characterized pathologically by chromogranin A (CgA) and synaptophysin (SYP) staining, with the former being more specific and the latter being more sensitive [33]. SYP represents a family of proteins presented in synaptic vesicles that store and release classic neurotransmitters in both nervous system and neuroendocrine tumors (NETs) [34]. An upregulated expression of SYP reflects an activated synaptic machinery that may cause or enhance tumor innervation and growth [11,35,36,37]. It has been reported that SV2A can be used for pathological assessment of NED in NETs [38,39,40,41], interestingly, with a greater similarity to SYP than to CgA [38]. In addition, SV2A has been identified as the key membrane receptor for botulinum neurotoxin, which might be of therapeutic potential for treatment of tumors featuring NE phenotypes, including CRPC [11,35,36]. Furthermore, the expression of SV2A was also found in correlation with the ability of colorectal cancer stem cells to produce functional neurons [42], indicative of its role in cancer innervation. Among the three synaptic vesicle glycoprotein isoforms (SV2A, SV2B, and SV2C), SV2A is the most dominant one found in cancers [43,44]. Unlike CgA present in both blood and tumors [45], SV2A and SYP are confined to innervated tumors, which is a desired feature for oncological imaging. By extracting SV2A genomic data in clinical CRPC and NEPC specimens from the available gene expression repositories, we found that SV2A gene expression was significantly elevated. Given that several SV2A specific radiotracers (e.g.,^11^C-UCB-A [46], ^11^C-UCB-J [47], ^18^F-UCB-H [48], and ^18^F-SDM-8/SynVesT-1/2 [49,50]) have been reported for synaptic density PET imaging of neurodegenerative diseases [51,52], we performed a PET imaging evaluation in an NEPC xenograft model using ^18^F-SynVesT-1, after having confirmed SV2A protein expression in NEPC cell lines and mouse xenografts through a comparative study.

## 2. Results

### 2.1. SV2A Gene Expression in Clinical Tumor Tissues: NEPC vs. CRPC-Adeno

**Figure 1:**
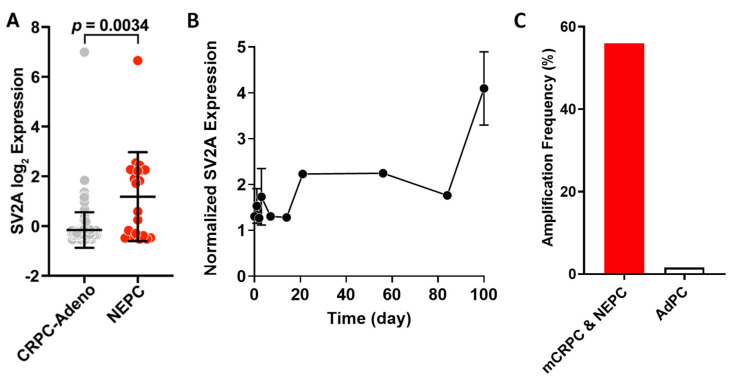
Elevated SV2A expression in NEPC tumors. (**A**) Normalized RNA expression of SV2A gene in NEPC (*n* = 20) and CRPC-Adeno (*n* = 147) patient tumor specimens. (**B**) SV2A expression change as AdPC was progressing to NEPC in a PDX mouse model after castration (GSE59986 dataset, *n* = 1–2). (**C**) Frequencies of amplified SV2A gene expression in patient specimens of mCRPC and NEPC (54 out of 96 or 56%) vs. AdPC (8 out of 499 or 1.6%). Note: The data in (**A**) are presented as mean ± SD; the data in (**B**) are presented as the average of expression at each time point post-castration (*n* = 2 at day 1, 2, 3, and 100; *n* = 1 at all other time points).

Our bioinformatic analyses were performed on combined data from three sources: a recent report by Beltran et al. [3], WCMC11/14, and SU2C 2015. We found that the normalized RNA expression of SV2A is significantly higher in NEPC (*n* = 20) than that in CRPC-Adeno (*n* = 147) (Figure 1A, *p* = 0.0034). Similar results were observed in another dataset (GSE104786, Supplementary Figure S1A). In addition, we observed an intriguing trend of SV2A expression in a patient-derived xenograft (PDX) model (GSE59986, Figure 1B) as AdPC was progressing to NEPC after castration; the SV2A expression maintained at a relatively stable and low level within 12 weeks post-castration until the emerging of the terminally differentiated NEPC, resulting in a drastic increase. Furthermore, we found that the frequency of SV2A gene amplification in mCRPC and NEPC samples (54 out of 96 or 56%, dataset by Beltran et al. [3]) was markedly higher than that in AdPC samples (8 out of 499 or 1.6%, TCGA Firehose Legacy dataset) (Figure 1C). As anticipated, NEPC samples also present higher expression levels of other neuronal markers in addition to SV2A than CRPC-Adeno samples (Supplementary Figure S1B,C). Taken together, SV2A expression is significantly elevated in NEPC and we reason it may serve as a potential target to develop efficacious theranostics for NEPC and other innervated cancers.

### 2.2. SV2A Protein Expression in PCa Cell Lines and Patient Tumor Specimens

**Figure 2:**
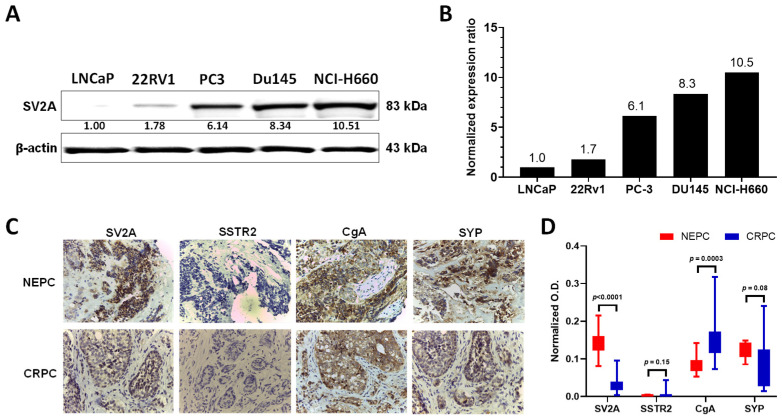
SV2A protein expression analyses. (**A**) Western blotting of SV2A in PCa cell lines (non-NEPC: LNCaP, 22Rv1; NEPC: PC-3, DU145, NCI-H660). β-actin was included as loading control. (**B**) Normalized expression of SV2A in each cell line to that in 22Rv1 set at 1.0. (**C**) Immunohistochemistry (IHC) staining of patient tissue samples representative of NEPC (*n* = 3) and CRPC (*n* = 7) against SV2A, SSTR2, CgA, and SYP (400×). (**D**) Quantitative analysis of IHC staining intensity using ImageJ 1.8.0.

Prompted by the encouraging results from the bioinformatic analyses, we did a comparative assay of SV2A protein expression in multiple PCa cell lines, including non-NEPC cell lines (LNCaP and 22Rv1) and NEPC lines (DU145, PC-3, NCI-H660). As expected, all NEPC cell lines exhibited markedly higher SV2A expressions than the non-NEPC cell lines (Figure 2A,B). Specifically, NCI-H660 had about a 23-fold higher SV2A expression than 22Rv1. Of note, SV2A is detectable in AdPC cell lines, likely indicating the early existence of NED and the proneness to further NED progression upon treatment. Indeed, when 22Rv1 cells were maintained under hypoxia for a few days, neurite-like structures became to emerge along with the elevated NED marker of neuron-specific enolase (NSE) [53]. In addition, 22Rv1 xenografts were found expressing NED markers within highly hypoxic tumorigenic regions [54].

We were able to obtain and analyze a limited number of NEPC (*n* = 3) and CRPC (*n* = 7) patient tissue samples (Figure 2C,D). Similar to SYP and CgA, SV2A showed strong immunohistochemistry (IHC) staining in NEPC samples, but with variations in CRPC samples. In contrast, it is noteworthy that the expression of SSTR2, the molecular target of ^68^Ga-DOTATATE, showed expressions in CPRC but became absent in NEPC.

### 2.3. SV2A Localization in PCa Cells

**Figure 3:**
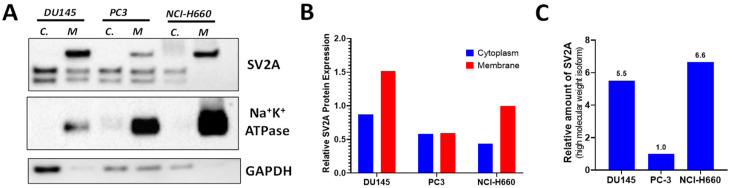
Subcellular localization of SV2A. (**A**) Western blotting assays of SV2A protein performed in the cytoplasmic (*C*) and membrane (*M*) fractions of PCa cells (DU145, PC3, and NCI-H660). Na^+^K^+^ ATPase subunit 1 staining reflects the membrane fractionation. (**B**) Relative SV2A protein expression in the cytoplasmic and membrane fractions of PCa cells. (**C**) Relative amount of the high molecular weight isoform of SV2A at cell membrane. For comparison, the amount in PC-3 is set at 1.0.

To determine the location of SV2A in PCa cells, we extracted cell membrane and cytoplasmic fractions from NCI-H660 cells. Immunostaining of subcellular fractionation demonstrated that SV2A predominately presents in the membrane rather than in the cytoplasm (Figure 3A). Of note, we observed that the membrane-bound SV2A isoform possesses a higher molecular weight than its cytoplasm counterpart, perhaps indicative of its higher extent of post-translational modifications. Given that the oncological version of SV2A-targeted agents likely lacks the desired cell membrane permeability for the brain imaging agent, ^18^F-SynVesT-1, clearly, these results further support that SV2A protein can be used for the development of targeted theranostics of NEPC.

### 2.4. Automated Radiosynthesis of ^18^F-SynVesT-1 on GE TRACERlab FX-N Synthesizer

Our previously reported radiosynthesis of ^18^F-SynVesT-1 was carried out in a semi-automated manner with ~19% of isolated yields (decay-uncorrected), in which the ^18^F-fluoride was loaded onto and eluted off a PS-HCO_3_ cartridge using a reversed flow elution [49]. Our initial attempts to fully automate the radiosynthesis on a GE TRACERlab FX-N synthesizer resulted in low yields (~5%) due to the low recovery (~35% vs. >90% semi-automated) of ^18^F-fluoride from the PS-HCO_3_ cartridge. In addition, it was hard to implement the reversed flow elution on the synthesizer without extra hardware modifications. Therefore, we applied a low-vacuum reaction vessel (~40 kPa pressure) to slowly drain the elution mixture through the cartridge. This modification allowed the low amount of K_2_CO_3_ solution to thoroughly interact with the anion-exchange resin, thus reproducibly improving the ^18^F-fluoride recovery over 95% (>3 runs) for the following radiolabeling procedures. The automated radiosynthesis of ^18^F-SynVesT-1 afforded ~ 15% of the isolated yield (decay-uncorrected; *n* > 3) with > 99% of radiochemical purity, similar to what we previously reported [49]. The total automated radiosynthesis time was 95–100 min.

### 2.5. PET Imaging of SV2A with ^18^F-SynVesT-1 in NEPC Xenograft Model

**Figure 4:**
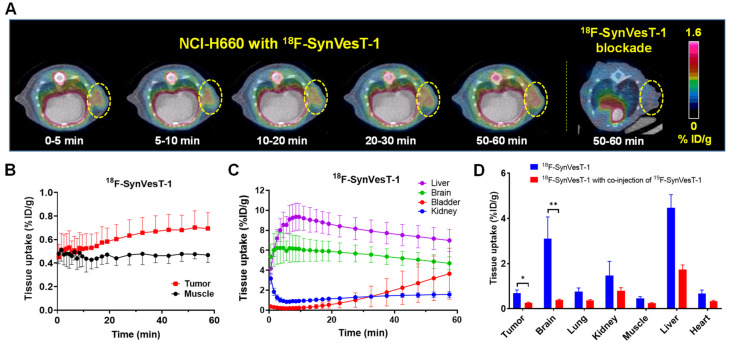
Mouse PET/CT imaging of SV2A with ^18^F-SynVesT-1. (**A**) Representative axial images in SV2A^+^ NCI-H660 tumor at five different time points and NCI-H660 tumor blocked with ^19^F-SynVesT-1. Tumors indicated by yellow circles. (**B**) Time-activity curves (TAC) of ^18^F-SynVesT-1 in NCI-H660 tumors and muscle. (**C**) TAC of ^18^F-SynVesT-1 in the liver, brain, bladder, and kidney. (**D**) Comparative uptake of ^18^F-SynVesT-1 and ^18^F-SynVesT-1 with co-injection of ^19^F-SynVesT-1 in tissues of interest from the imaging studies in NCI-H660. For clarity, only the data at 50–60 min is presented. * *p* = 0.025; ** *p* = 0.0082. The data are presented as mean ± SD, *n* = 3.

Given the significantly elevated SV2A expression in NEPC cells and xenografts, we performed a pilot small animal PET/CT imaging evaluation with ^18^F-SynVesT-1 in NOD-SCID mice bearing NCI-H660 xenografts. We acknowledge that although ^18^F-SynVesT-1 has entered clinical trials [41,51], it is a neuroimaging agent that targets SV2A in the brain for synaptic density assessment but not in NEPC for oncological imaging. Despite the undesired high hepatic accumulation due to its lipophilic nature, ^18^F-SynVesT-1 was able to clearly visualize the SV2A^+^ NCI-H660 tumor within 1 h p.i. (Figure 4A and Supplementary Figure S2A). The quantitative data analysis of the dynamic PET imaging displayed that the uptake of ^18^F-SynVesT-1 in the tumor was consistently higher than that in the muscle, starting from 10 min p.i. It maintained a stable and increasing trend within 60 min p.i., peaked at 0.70 ± 0.14 %ID/g in between 50–60 min p.i., and then gradually decreased to 0.29 ± 0.04 %ID/g at 4 h p.i. (Figure 4B and Supplementary Figure S3A,B). Within the 4 h of imaging, the uptake of ^18^F-SynVesT-1 stayed virtually unchanged in the muscle. As such, the highest tumor-to-muscle ratio was 1.47, observed in between 50–60 min p.i. (Supplementary Figure S3C). To confirm the SV2A-specific uptake of ^18^F-SynVesT-1 in the NCI-H660 tumor, we performed the blocking study with a co-injection of ^19^F-SynVesT-1 (0.1 mg, 0.033 µmol). The uptake of ^18^F-SynVesT-1 in NCI-H660 tumor was significantly reduced from 0.70 ± 0.14 %ID/g to 0.25 ± 0.03 %ID/g at 50–60 min p.i. (*p* = 0.025), the same level as in the muscle. At the same time, the brain uptake also showed a drastic decrease (*p* < 0.01) (Figure 4D), indicating the successful blockade of SV2A-specific uptake and the SV2A-specific binding of ^18^F-SynVesT-1 in the NCI-H660 tumor. As anticipated, ^18^F-SynVesT-1 showed high accumulation in both liver and brain (Figure 4C and Supplementary Figure S2A). The partial renal clearance of ^18^F-SynVesT-1 was reflected by the increasing uptake in the bladder (Supplementary Figure S2A).

**Figure 5:**
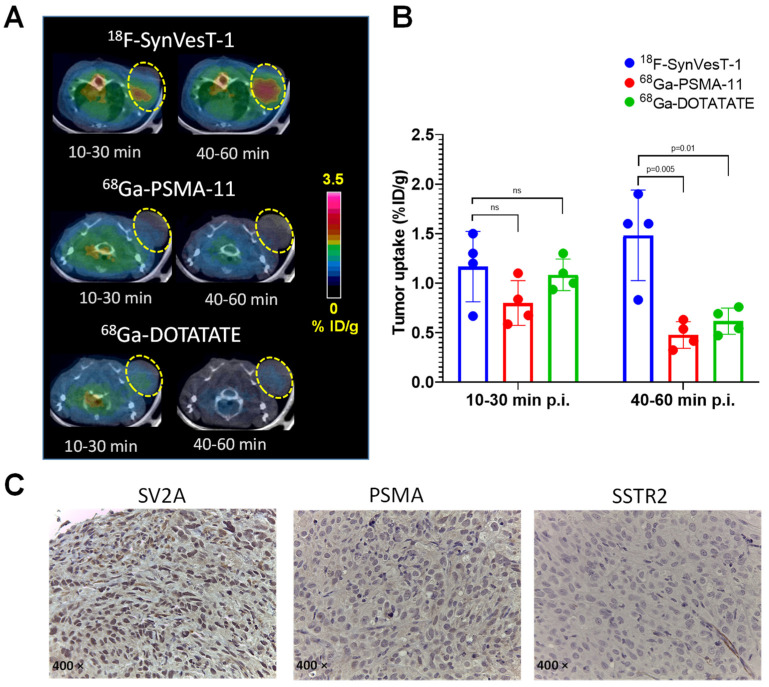
Comparative PET/CT imaging of SV2A with ^18^F-SynVesT-1, ^68^Ga-PSMA-11, and ^68^Ga-DOTATATE in mice bearing SV2A^+^ DU145 tumors. (**A**) Representative axial images in DU145 tumor presented within two different time frames for all three radiotracers. Tumors indicated by yellow circles. (**B**) Quantitative tumor uptake in DU145 tumors (*n* = 4; ns: not significant, *p* > 0.05). (**C**) IHC staining of SV2A, PSMA, and SSTR2 in the harvested DU145 tumors.

To demonstrate the diagnostic value of PET imaging of SV2A for noninvasive assessment of NED in NEPC, we carried out another set of small animal PET/CT imaging evaluations with ^18^F-SynVesT-1, in direct comparison with ^68^Ga-PSMA-11 (targeting PSMA) and ^68^Ga-DOTATATE (strong binding to SSTR2 [55]) in NOD-SCID mice bearing the NEPC DU145 xenografts. Similar to the observations in the NCI-H660 xenograft model, DU145 tumors were clearly visualized by ^18^F-SynVesT-1 (Figure 5A), with the uptake at 1.17 ± 0.36 %ID/g (10–30 min p.i.) and 1.48 ± 0.46 %ID/g (40–60 min p.i.) (Figure 5B). In contrast, neither ^68^Ga-PSMA-11 nor ^68^Ga-DOTATATE displayed meaningful tumor-to-background uptake ratios (Figure 5A). Although their tumor uptake values (0.80 ± 0.23 %ID/g and 1.08 ± 0.16 %ID/g, respectively) were at a similar level to that of ^18^F-SynVesT-1 within 10–30 min p.i., a significant reduction was observed for both agents (*p* ≤ 0.01) within 40–60 min p.i. It indicated the retention of ^18^F-SynVesT-1 was SV2A-specific in the tumors, while the tumor accumulation of ^68^Ga-PSMA-11 and ^68^Ga-DOTATATE was not related to NED (Figure 5B). Immediately after the imaging, the DU145 tumors were harvested for IHC staining of SV2A, PSMA, and SSTR2. Shown in Figure 5C, the tumors displayed clearly positive staining of SV2A but not of PSMA or SSTR2, which further validates the PET imaging observations (Figure 5A,B).

## 3. Discussion

The extent of NED in PCa has been recognized, with an essential role in the prognosis of patients with PCa [56]. The lack of an imaging method for noninvasive assessment of NED in PCa and other cancer types represents not only a major unmet clinical need in the current cancer patient care but also an opportunity to leverage this biomarker for developing novel cancer theranostics.

Given the clinical rarity of NEPC specimens and our experience in SV2A imaging, we started our work with bioinformatic analyses, using the reported PCa databases where SV2A had been profiled but not analyzed in detail (Figure 1). It is noteworthy that there were only 20 NEPC specimens available in the analysis and their gene expression levels of SV2A varied in a wide range. However, we found that SV2A gene expression is indeed upregulated in CRPC and further amplified in NEPC. As such, we performed a screening for SV2A in a variety of PCa cell lines, non-NEPC and NEPC (Figure 2). Not surprisingly, we observed that higher SV2A expressions are in association with NEPC cell lines.

Earlier studies on NETs and neuroblastoma [40,43] reported that the higher molecular weight isoform of SV2A was presented in the cytoplasm. However, in the PCa cell lines assayed in this work, we found that it is predominately cell membrane-bound instead (Figure 3), which is of advantage when considering SV2A as a target for the design and development of cancer theranostics.

To provide a proof of concept, we performed an imaging evaluation of SV2A using ^18^F-SynVesT-1 in NEPC xenograft mouse models established by NCI-H660 and DU145. Even with the neuroimaging agent that targets SV2A in the brain for synaptic density assessment but not SV2A in NEPC for oncological imaging [41,51], we were able to clearly visualize the SV2A^+^ tumors and confirm the desired SV2A-imaging specificity (Figure 4) for noninvasive assessment of NED in NEPC or other innervated cancer types. Clinically, NEPC features reduced expressions of PSMA, while the expressions of SSTRs (1–5) in NEPC remain inconclusive. Indeed, the results of our work presented herein demonstrated the limited diagnostic value of PET imaging with ^68^Ga-PSMA-11 or ^68^Ga-DOTATATE for the detection of NED in NEPC.

Because of the lipophilic nature of ^18^F-SynVesT-1, which is required for a neuroimaging agent to cross the blood–brain barrier (BBB), we observed its high uptake and retention in the brain, liver, and intestines. The high accumulation of the agent in the abdominal region is of less concern for brain imaging but it presents a likely insurmountable obstacle for PCa imaging. Of note, the high brain uptake may also present a challenge for neuro-oncological imaging of SV2A due to the inevitable high presence of SV2A in the brain. We also observed an appreciable level of renal excretion of ^18^F-activity, likely resulting from the less lipophilic metabolites of ^18^F-SynVesT-1. These metabolites might not be able to cross the BBB to adversely affect the brain imaging, but they may complicate the image interpretation and data quantification when using the agent for cancer imaging, particularly for PCa. Therefore, although our proof-of-concept data is promising, we must re-design the SV2A-targeted brain imaging probes for oncological applications.

While maintaining the high affinity and specificity to SV2A, the oncological SV2A agents must have a substantially different in vivo kinetic distribution profile from their neuroimaging counterparts. For neuroimaging, in general, a lipophilicity measured by logP from 2 to 4 is required for optimal passive brain entry [57,58,59]. The measured logP value of ^18^F-SynVesT-1 (2.32) is right in the range [49]. Due to the lipophilicity, the plasma free fraction (*f*p) of ^18^F-SynVesT-1 is ~43 ± 2% [49], which means ~57% of the radiotracer stays protein-bound in the blood. For oncological applications, the SV2A targeting agents will have to be modified to be hydrophilic so that their *f*p values can be high for effective cell membrane SV2A binding and rapid renal clearance.

Although this work focuses on providing a proof-of-concept for SV2A-targeted PET imaging of NED in PCa, it can certainly be extended to other innervated cancer types beyond PCa. For instance, somatostatin receptor-based PET imaging is routinely practiced in NETs [60], but it is most useful in well-differentiated gastroenteropancreatic NETs (GEP-NETs) [61,62,63,64]. In contrast, SV2A has been reported with high expressions in a broad spectrum of cancer types with NED [41,43,65]. Therefore, SV2A can be potentially used to develop early detection methods for NED, which will provide new insights into the diagnosis and treatment of cancers with NED features, and theranostic agents for innervated cancers.

## 4. Materials and Methods

### 4.1. General

All reagents and solvents were purchased from commercially available sources and used as received unless otherwise stated. All aqueous solutions were prepared with Milli-Q water (18 MΩ-cm), which was obtained from a Millipore Gradient Milli-Q water system.

### 4.2. Bioinformatic Analyses Data Source

The SV2A gene expression data in NEPC patient tumors (WCMC11/14, SU2C 2-15, and Beltran et al. [3]) were downloaded from the cBioPortal database (http://www.cbioportal.org, accessed on 19 February 2019). The data from “The Expression data from Neuroendocrine Prostate Cancer and Primary Small Cell Prostatic Carcinoma” (GSE104786) and NEPC patient-derived xenograft (PDX) study (GSE59986) were downloaded from the Gene Expression Omnibus repository (https://www.ncbi.nlm.nih.gov/gds, accessed on 31 January 2020). The frequency of increased SV2A gene copy number was analyzed by the built-in analysis tool in the cBioPortal website for the comparative assay of two sets of data: the NEPC data from the cBioPortal database vs. the PCa data from the Cancer Genome Atlas (TCGA) database, linked through the cBioPortal website. Prism 8.0 (GraphPad Inc., San Diego, CA, USA) was used to run statistical analyses and plot the graphs.

### 4.3. Cell Culture and Animal Models

All animal studies were performed in accordance with relevant guidelines and regulations through an animal protocol (APN: 2020-102858; effective from 26 May 2020 to 26 May 2023) approved by the Institutional Animal Care and Use Committee (IACUC) at UT Southwestern, which adheres to the ARRIVE guidelines. Human LNCaP, 22Rv1, PC-3, DU145, and NCI-H660 PCa cell lines were obtained from the American Type Culture Collection (ATCC). The NCI-H660 cells were cultured in a complete growth medium according to the culture method supplied by ATCC. 22Rv1 cells were cultured in DMEM media with 10% fetal bovine serum (FBS) and 1% Penicillin/Streptomycin (PS). Other cells were cultured in RPMI media with 10% FBS and 1% PS. All cells were cultured in a humidified chamber at 37 °C with 5% CO_2_.

To generate the mouse subcutaneous tumor models, DU145 cells (1 × 10^6^ cells in 100 μL of 1:1 (*v*/*v*) matrigel and phosphate-buffered saline (PBS) were injected subcutaneously into the right shoulder of non-obese diabetic-severe combined immunodeficiency (NOD-SCID) mice; 22Rv1 cells (2.5 × 10^6^ cells in 100 μL of 1:1 (*v*/*v*) matrigel and PBS) were injected subcutaneously into the shoulders of NOD-SCID mice; NCI-H660 cells (5.0 × 10^6^ cells in 100 μL of 1:1 (*v*/*v*) matrigel and PBS) were injected subcutaneously into the right shoulder of NOD-SCID mice. After injection, mice were monitored 1–2 times a week. The tumor volume was determined by the formula of Volume = (Width^2^ × Length)/2.

### 4.4. Western Blotting

Cells were washed with PBS buffer and lysed in RIPA buffer (50 mM Tris buffer pH 8.0, 150 mM NaCl, 0.1% SDS, 1% NP-40, and 0.5% sodium deoxycholate) containing protease inhibitors. Collected proteins were loaded onto Bolt 4–12% Bis-Tris Plus gel (Invitrogen). After 60 min of electrophoresis (110 V) at room temperature (RT), they were transferred to a nitrocellulose membrane. The membrane was blocked with 5% skim milk at RT for 1 h and then incubated with primary antibodies (Rabbit anti-human SV2A (1:1000), Sigma catalog #HPA007863; mouse anti-GAPDH (1:2000), Santa Cruz, sc-47724) at 4 °C overnight, followed by washing with Tris-buffered saline with 0.1% Tween^®^ 20 detergent (TBST) solution, and 1 h incubation with horseradish peroxidase-conjugated secondary antibodies (Peroxidase-AffiniPure Goat Anti-Rabbit IgG (H + L), Jackson ImmunoResearch catalog #111-035-144; Peroxidase-AffiniPure Goat Anti-Mouse IgG (H + L), Jackson ImmunoResearch catalog #115-035-146) at RT. Protein bands were visualized by a Molecular Imager ChemiDoc XRS System (Bio-Rad Laboratories, Hercules, CA, USA). Densitometry of various proteins and their respective loading controls from the same blot was performed using ImageJ 1.8.0 (NIH) software. Relative density was calculated by dividing the densitometry of protein with the loading control.

### 4.5. Determination of the SV2A Location in PCa Cells

Membrane and cytoplasmic fractions were extracted using a subcellular protein fractionation kit for cultured cells (78840, Thermo Scientific, Waltham, MA, USA) per manufacturer’s protocol. The fractions were then subjected to gel electrophoresis and blotted. Anti-SV2A antibody (ab32942, Abcam, Waltham, MA, USA) was used at a dilution of 1:1000, and Anti-rabbit IgG, HRP-linked Antibody, Cell Signaling Technology #7074, was used as the secondary antibody; GAPDH antibody (sc-166574, Santa Cruz Biotechnology, Inc., Dallas, TX, USA) was used at a dilution of 1:2000, and Anti-mouse IgG, HRP-linked Antibody (#7076, Cell Signaling Technology, Danvers, MA, USA) was used as the secondary antibody; and NaK ATPase subunit 1 antibody (3010S, Cell Signaling Technology, Danvers, MA, USA) was used at a concentration of 1:500. Specificity for membrane fractionation was evaluated by blotting for NaK ATPase subunit 1, while specificity for cytoplasmic fractionation was evaluated by blotting for GAPDH. Unstained gel and membrane were imaged with the ChemiDoc MP Imaging system (BioRad Laboratories, Hercules, CA, USA). Protein quantification was done via analyzing relative intensities of each band using ImageJ. Both the top membrane-specific SV2A and the total SV2A protein were normalized to protein levels in the unstained gel. The quantification was assessed via densitometry analysis.

### 4.6. Automated Radiosynthesis of ^18^F-SynVesT-1

The preparation of ^18^F-SynVesT-1 was conducted in a GE TRACERlab FX-N synthesis module (GE Healthcare, Waukesha, WI, USA) with a modification from the reported procedure [49]. Briefly, the cyclotron-produced ^18^F-fluoride was trapped in a pre-conditioned Chromafix PS-HCO_3_ cartridge, through which a mixture of KOTf in Milli-Q water (10 mg/mL, 0.45 mL), K_2_CO_3_ in Milli-Q water (1 mg/mL, 50 μL), and MeCN (0.5 mL), was pulled into a low vacuum reaction vessel (~40 kPa pressure to start the drainage). The following radiochemical procedures were performed as reported [49].

### 4.7. Small Animal PET/CT Imaging

The imaging study was started when the tumor size reached 200–500 mm^3^ on a Inveon PET/CT Multimodality System (Siemens Medical Solutions USA, Inc.,Knoxville, TN, USA). Followed by CT data acquisition, which was conducted at 80 kV and 500 μA with a focal spot of 58 μm, a dynamic scan (0–60 min) was performed immediately after intravenous injection of ~3.5 MBq of ^18^F-SynVesT-1 in 100 μL saline containing < 5% ethanol into each tumor-bearing mouse under anesthesia, with 2% isoflurane in oxygen. After the dynamic scan, the mouse was allowed to recover in a cage and then re-anesthetized for a 20 min scan at 2.5 and 4 h post-injection (p.i.) (at each time point, *n* = 3–4). Both CT and PET images were reconstructed with the manufacturer’s software. Reconstructed CT and PET images were fused for quantitative data analysis. Regions of interest (ROIs) were drawn as guided by CT and quantitatively expressed as percent injected dose per gram of tissue (%ID/g).

### 4.8. Immunohistochemistry (IHC)

All xenograft and patient tumor specimens were fixed in 10% formalin and embedded in paraffin, then IHC was performed on 5 µm sections. Briefly, sections were first deparaffinized and rehydrated, and then antigens were retrieved in citrate buffer. After blocking the endogenous peroxidase with 0.3% hydrogen peroxide for 20 min and proteins with 5% serum for 1 h, sections were incubated overnight at 4 °C with 1:400 diluted primary antibody against SV2A (HPA007863, Sigma -Aldrich, St. Louis, MO, USA), SSTR2 (ab134152, Abcam, Waltham, MA, USA), SSTR5 (ab28618, Abcam, Waltham, MA, USA), PSMA (ab19071, Abcam, Waltham, MA, USA), CgA (ab45179, Abcam, Waltham, MA, USA), or SYP (ab32127, Abcam, Waltham, MA, USA). Next, the sections were incubated with biotinylated anti-rabbit or anti-mouse secondary antibodies (#BA-1100 and #BA-2000, Vector Laboratories Burlingame, CA, USA) at room temperature for 1h. After washing for 10 min three times in PBS, the sections were incubated with ABC-HRP complex (#PK-4000, Vector Laboratories Burlingame, CA, USA) for 1 h. Then slides were developed with 3,3′-diaminobenzidine (SK-4105, Vector Labs, Burlingame, CA, USA). All the slides were mounted after counterstaining with hematoxylin. Staining signals were photographed at high-power fields (×40) using an Olympus BX51 Microscope (Olympus, Tokyo, Japan).

### 4.9. Statistical Analysis

Statistical analyses were performed using GraphPad Prism 8.0. A *p*-value less than 0.05 (unpaired *t*-test) was considered statistically significant. All quantitative data are presented as mean ± standard deviation.

## 5. Conclusions

In this work, high SV2A expressions were found in association with NED in CRPC and NEPC by a set of bioinformatics analyses performed on available databases. Further screening and assays of PCa cell lines confirmed the upregulated SV2A in the NEPC cell lines and that the high molecular weight SV2A isoform is predominately cell membrane-bound. The specific SV2A imaging with ^18^F-SynVesT-1 in an NEPC xenograft model provided the proof of concept and laid the foundation for oncological applications of SV2A-targeted theranostic agents.

## Data Availability

Data are available in this manuscript or from authors upon reasonable request.

## Supplementary Materials

The following are available online at https://www.mdpi.com/article/10.3390/ijms222313085/s1.
