# Supplementary material for: Validation of SV2A-Targeted PET Imaging for Noninvasive Assessment of Neuroendocrine Differentiation in Prostate Cancer
Source: Int J Mol Sci. 2021 Dec 3;22(23):13085. doi: 10.3390/ijms222313085 (PMC8657802; doi:10.3390/ijms222313085)
Supplement: Supplementary file 1 [file ijms-22-13085-s001.zip › ijms-1460191-supplementary.pdf]

SUPPLEMENTARY INFORMATION

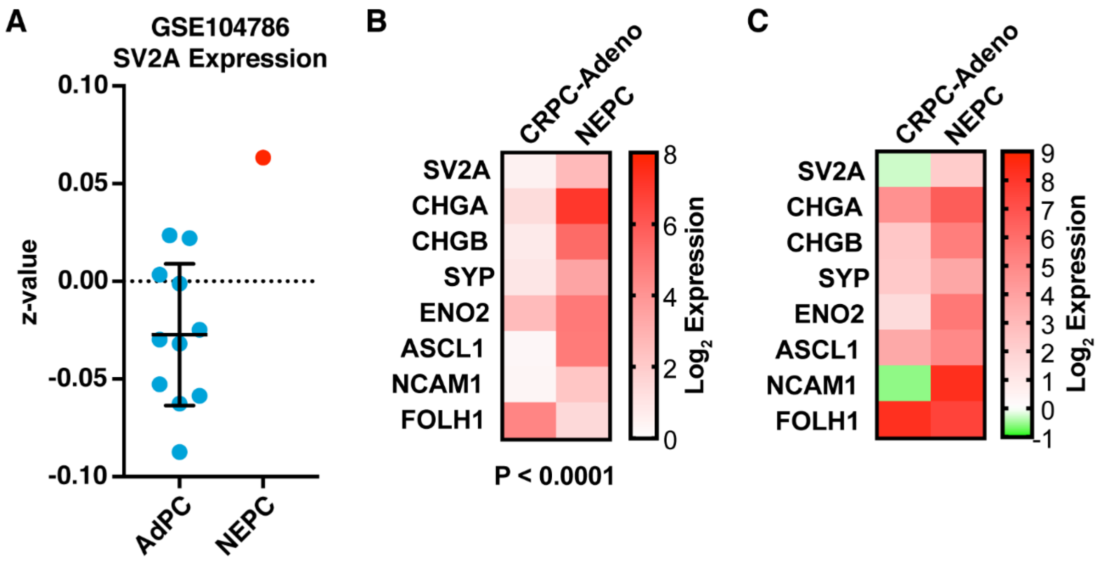

Supplementary Figure S1. The expression of SV2A is elevated with other neuronal biomarkers in the NEPC patient tumors. (**A**) The RNA expression of SV2A is higher in the NEPC patient tumors (*n* = 1) compared to the AdPC patient tumors (*n* = 11) in the GSE104786 study. (**B**) The expressions of neuronal biomarkers including SV2A are higher in the NEPC patient tumors (*n* = 43) than those in the CRPC-Adeno (*n* = 53) patient tumors in the combined study published in Beltran et. al in 2016. (**C**) A similar trend of elevated expressions of SV2A and other neuronal biomarkers in NEPC patient tumors was observed in SU2C 2015 study (CRPC-Adeno, *n* = 113; NEPC, *n* = 5). SV2A, Synaptic vesicle glycoprotein 2A; CHGA, Chromogranin A; CHGB, Chromogranin B; SYP, Synaptophysin; ENO2, Neural enolase; ASCL1, Achaete-Scute family BHLH transcription factor 1; NACM1, CD56; FOLH1, Folate hydrolase 1, which is the gene that encodes prostate-specific membrane antigen (PSMA).

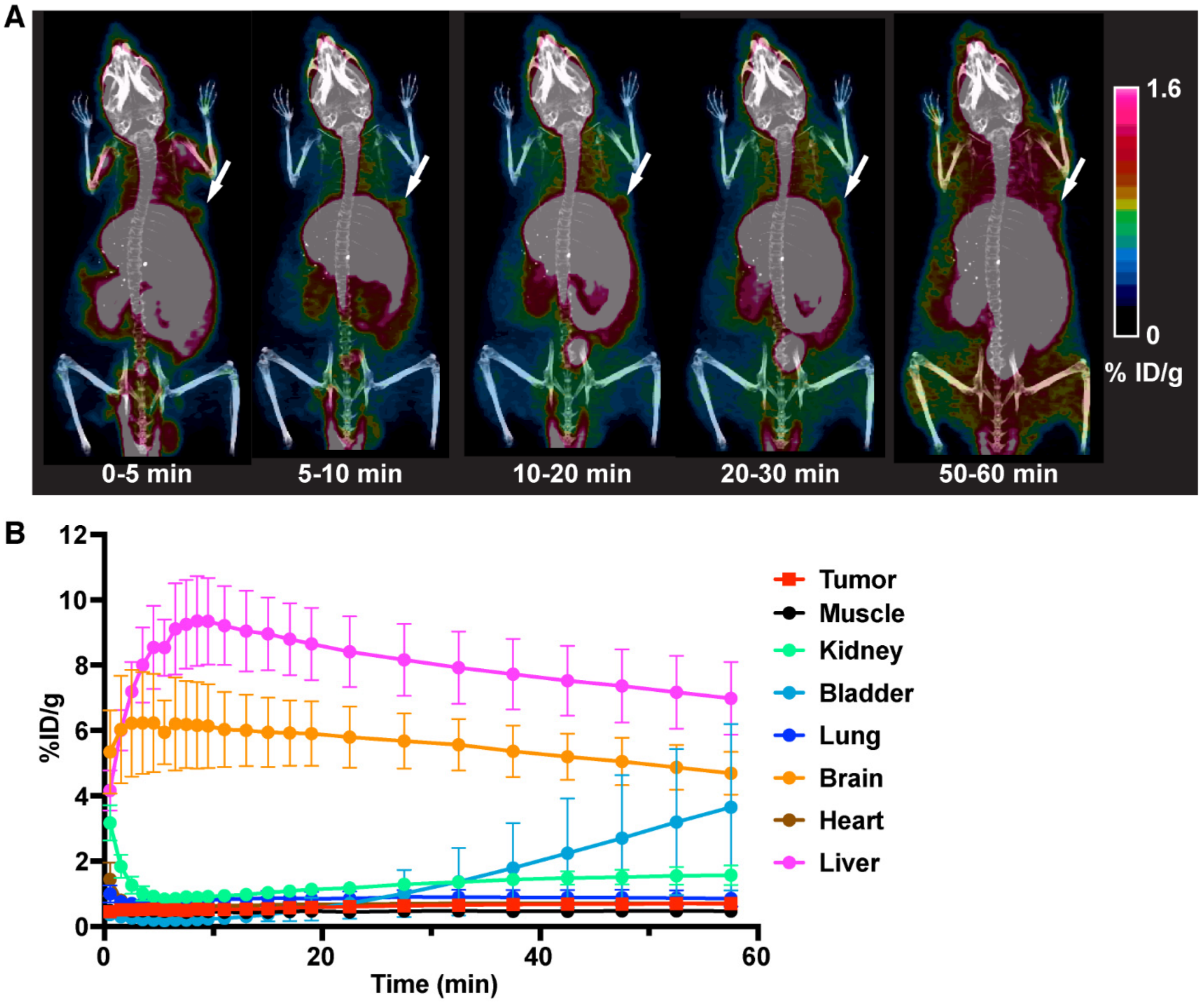

Figure S2. The time–activity curves of ^{18}F-SynVesT-1 uptake in tissues/organs in the NOD-SCID mice bearing subcutaneous NCI-H660 tumor. (**A**) The representative ^{18}F-SynVesT-1 PET images of NIC-H660 tumor-bearing NOD-SCID mouse. The location of tumor is indicated by white arrows. (**B**) The plot demonstrates the TAC curves of ^{18}F-SynVesT-1 in tissues monitored in the first 60 min post injection of ^{18}F-SynVesT-1. The data is presented as mean ± SD (n = 3).

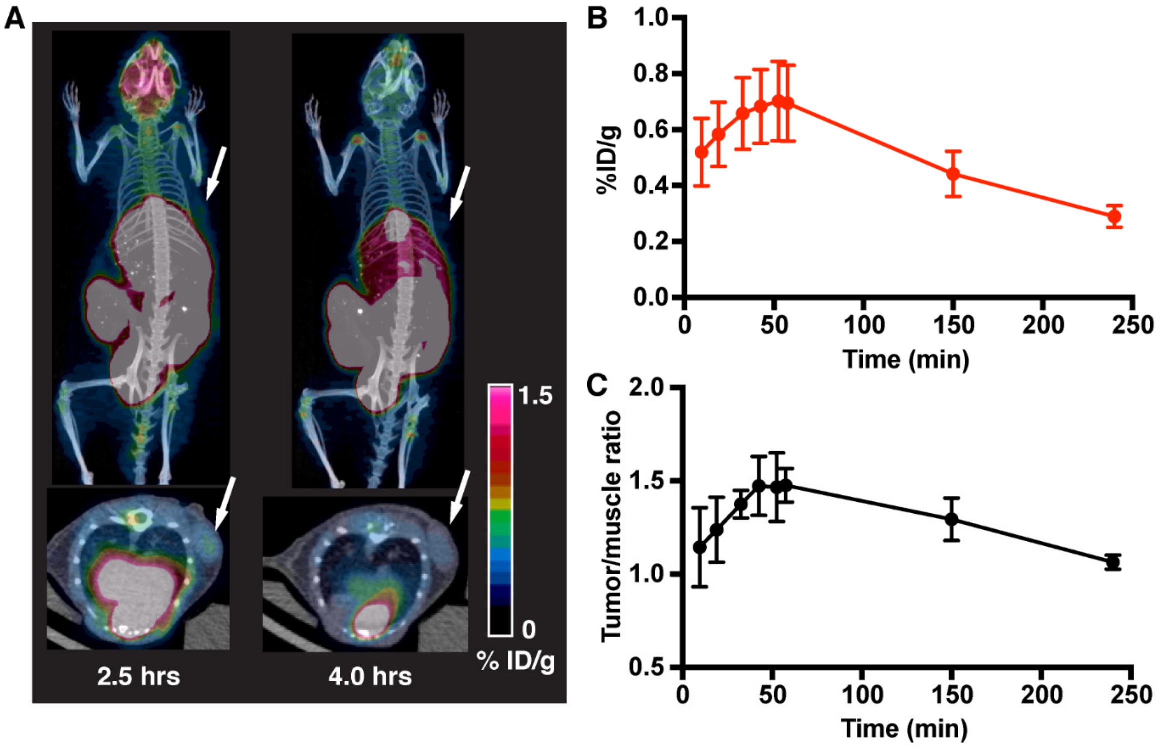

Figure S3. The time–activity curve of ^{18}F-SynVesT-1 uptake in NCI-H660 subcutaneous tumor in NOD-SCID mice up to 4 h. (**A**) Representative ^{18}F-SynVesT-1 PET images from the same NCI-H660 tumor-bearing mouse at 2.5 h (left) and 4.0 h (right) post injection of ^{18}F-SynVesT-1. The tumor location is indicated by white arrow. (**B**) The TAC of ^{18}F-SynVesT-1 uptake in NCI-H660 subcutaneous tumor up to 4 h p.i. (**C**) The tumor/muscle ratio curves along time up to 4 h p.i. The data is presented as mean ± SD (n = 3).
